# Evaluation of the efficiency of corticosteroids in treating adult with sepsis: A protocol for systematic review and meta-analysis

**DOI:** 10.1097/MD.0000000000025610

**Published:** 2021-05-07

**Authors:** Ke-Peng Li, Yan-Yan Zeng, Li-Li Li, Wan-Peng Wang

**Affiliations:** a Department of Critical Care Medicine, Weifang People's Hospital, Weifang; b Department of Chronic Disease Prevention, Rizhao Center for Disease Control and Prevention, Rizhao; c Department of Infectious Diseases, Weifang People's Hospital, Weifang, Shandong Province, China.

**Keywords:** corticosteroids, efficiency, meta-analysis, sepsis

## Abstract

**Background:**

Sepsis is a worldwide health problem that is a leading cause of mortality due to infection. Sepsis is prevalent in infections that are complicated with organ failure. Generally, sepsis is intricate and impaired corticosteroid metabolism leads to complex outcomes. Therefore, the provision of corticosteroids could lead to improved clinical outcomes. The effect of corticosteroids therapy in adult patients with sepsis is not well studied. Therefore, this study is an attempt to evaluate the efficacy of corticosteroids for treating adult cases of sepsis.

**Methods:**

We will systematically search the randomized controlled trials for potential eligible studies from online databases, which includes 5 English databases (PubMed, EMBASE, Web of Science, PsycINFO, and Cochrane Library) and 4 Chinese databases (China National Knowledge Infrastructure, WanFang Database, VIP information database, and China Biomedical Database) from their origin to March 2021. Languages were restricted to English and Chinese. Two independent authors will be screening the literature, collect, and perform data extraction and quality assessment. Data will be synthesized using appropriate statistical methods.

**Results:**

This study will summarize present evidence to evaluate the efficacy of corticosteroids for the treatment of adult cases of sepsis.

**Conclusion:**

The results of the present study will provide the latest, reliable, superior quality evidence for the clinical application of corticosteroids for treating sepsis patients.

**Ethics and dissemination:**

The present study will use published data and does not require ethics approval.

**Protocol registration number:**

March 28, 2021.osf.io/tm6sw. (https://osf.io/tm6sw/)

## 1 Introduction

Sepsis is a disease with serious consequences. It is characterized by organ dysfunction due to an imbalance in host responses to bacteria, fungi, or viral infections.^[1]^ The dysregulated host response is usually defined by the presence of a successive organ failure evaluation score of 2 or more.^[1,2]^ The dysregulated response may result in systematic inflammation and organ damage, or in immune paresis and secondary infection.^[3,4]^ According to a previous research, adult patients were admitted to 409 academic, communal, and centralized hospitals in the United States of America from 2009 to 2014, sepsis was prevalent in 6% of adult hospital admissions.^[5]^ The lack of diagnostic test for sepsis is a main problem faced by sepsis patients, as it is one of the leading causes of death for patients admitted to the intensive care unit. Typically, such patients pass away from hypotension or successive multiple organ failures.^[6–9]^ Epidemiological survey shows that an aging population, widespread use of chemotherapy and immunosuppressive therapy has increased the global incidence of sepsis patients. Consequently, the prevalence of sepsis is a significant financial burden on the healthcare system.^[9,10]^

Corticosteroids comprise natural steroid hormones secreted by adrenocortical cells and an extensive range of synthetic analogues. These compounds have varying effects, these are roughly categorized as glucocorticoid and mineralocorticoid effects. Glucocorticoid effects primarily include the regulation of carbohydrates, lipids, protein metabolism, and inflammation regulation.^[11,12]^ Mineralocorticoid effects primarily include the regulation of electrolyte and water metabolism.^[11,12]^ Overall, the underlying molecular mechanisms of action of glucocorticoids are suitable for countering the hysterical inflammation could characterize sepsis. According to the author's best knowledge, this is the first trial aimed at attempting to find solutions to these questions regarding the suitability of such treatments. However, previous research has mostly been small-sample analysis without sufficient evidence to aid the clinical treatment of patients with sepsis using corticosteroids. Therefore, we will attempt to clarify the efficacy of corticosteroids as a treatment for adult cases of sepsis by conducting a meta-analysis of existing reports.

## 2 Methods

The present protocol is registered on the Open Science Framework (OSF, http://osf.io/) under the registration DOI number 10.17605/OSF.IO/TM6SW, and designed under the guidelines established by the Preferred Reporting Items for Systematic Reviews and Meta-Analysis Protocol (PRISMA-P) Statement.^[13]^

### 2.1 Inclusion and exclusion criteria

#### 2.1.1 Inclusion criteria

This study will include all the randomized controlled trials (RCTs) on corticosteroids that treat adult sepsis patients. Languages were restricted to English and Chinese.

#### 2.1.2 Exclusion criteria

Following studies will be excluded: repeated publications, case reports, letters, animal studies, and non-RCTs.

### 2.2 Types of participants

This study will include participants aged over 18 years. These adults should be diagnosed with sepsis caused by any micro-organism.

### 2.3 Types of interventions

The empirical intervention was all types of corticosteroid for treating sepsis. The control group was administered antibiotics, mechanical ventilation, fluid replacement, renal replacement therapy, or any other replacement therapy.

### 2.4 Types of outcome measures

The major clinical outcomes for this study include all-cause mortality, short-term mortality: mortality assessed on the 30th day, and long-term mortality: measuring mortality at time periods higher than 30 days. The minor clinical outcomes for this study entail organ failure development, bacteriological failure rate, length of hospitalization for survivors, fatalities due to septic shock, and adversities.

### 2.5 Information sources and search strategy

We will systematically search RCTs for potential eligible studies from electronic databases, which include 5 English databases (PubMed, EMBASE, Web of Science, PsycINFO, and Cochrane Library) and 4 Chinese databases (China National Knowledge Infrastructure, WanFang Database, VIP information database, and China Biomedical Database) from their beginning to March 2021. Additionally, other sources are also examined, such as ClinicalTrials.gov, the reference lists of all relevant studies, and gray literature, to include all potentially related articles. The following MeSH terms, related synonym, and their combinations will be searched in the above-mentioned databases: sepsis∗, corticosteroids∗, “adrenal cortex hormones,” glucocorticoids∗, “randomized controlled trial,” “randomised controlled trial,” randomly∗, and RCT∗.

### 2.6 Data collection and analysis

#### 2.6.1 Studies selection

**Figure 1:**
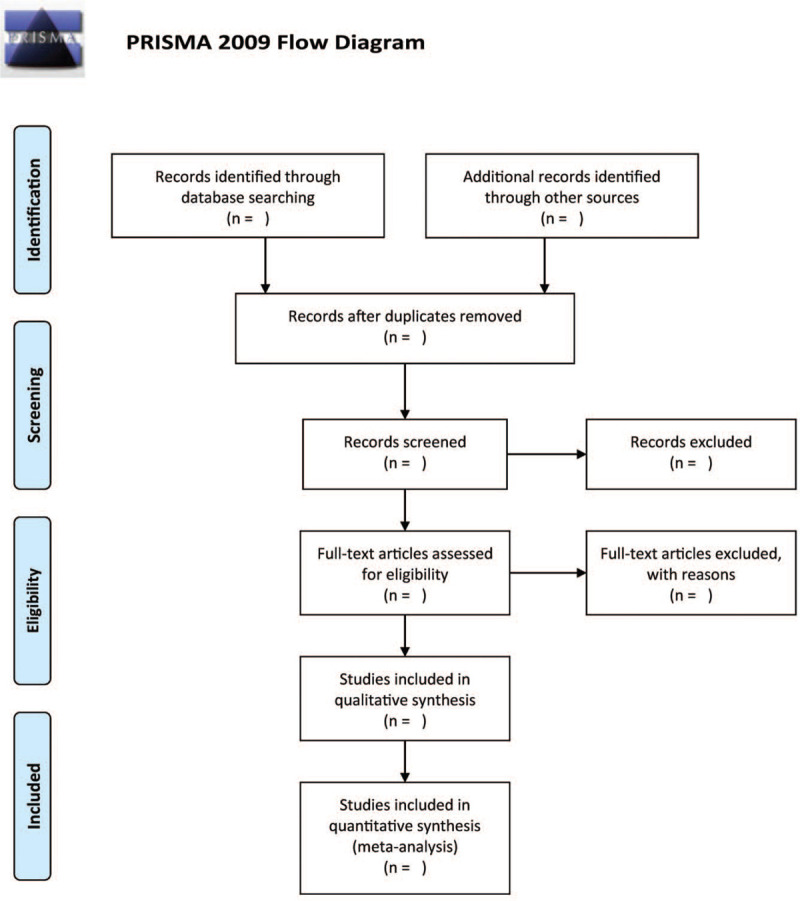
The research flowchart.

EndNote X9 will be applied to manage all searched records and to delete duplicated publications. Two independent authors will be screening titles/abstract of literature to eliminate irrelevant studies. Afterward, the complete text of each included study will be evaluated to decide whether they fulfil the inclusion criteria. All discrepancies are resolved via discussion or through consultation with a third author where necessary. The research flowchart is shown in Figure 1.

#### 2.6.2 Data extraction and management

A predesigned Excel 2019 table will be used by a couple of authors to extract the following information from included study: study characteristics: author, publication date, design of the study, and sample size; participant characteristics: age, gender, ethnicity, diagnostic criteria; interventions: intervention, comparing, alternative cointerventions; outcome measures: primary and secondary outcomes, obtaining, and reporting time points; and other crucial information. All disagreements shall be determined through discussion or by consulting a third author when necessary.

#### 2.6.3 Assessment of methodological quality

Two authors will independently evaluate the bias risk identified in the present review with the Cochrane Collaboration's “Risk of bias” assessment tool.^[14]^

#### 2.6.4 Measures of treatment effect

Dichotomous data will be expressed as the risk ratio together with 95% confidence intervals. Continuous data will be expressed as the mean difference or standardized mean difference together with 95% confidence intervals.

#### 2.6.5 Dealing with missing data

Any absent or unclear data will be requested from the corresponding author via email or phone.

#### 2.6.6 Assessment of heterogeneity

This study will employ I^2^ value to determine the heterogeneity. I^2^ < 50% is considered an indication of no obvious statistic heterogeneity, in which case the fixed-effects model is adopted to merge data;^[15]^ else, the random-effects model is utilized to merge data.^[16]^

#### 2.6.7 Sensitivity analysis

Low-quality studies or insufficient sample size studies will be eliminated to evaluate the stability of our findings.

#### 2.6.8 Assessment of reporting biases

This study will carry out funnel plots to evaluate any potential reporting bias.

## 3 Discussion

Generally, sepsis remains as a condition that inflicts high morbidity and mortality rate in the rescue and treatment of patients in critical state.^[1]^ Admittedly, with the development of medical treatment, there has been progress with regards to understanding and treating sepsis. However, the prognosis of sepsis is still not optimistic.^[17]^ Some published studies have found that the application of corticosteroids holds a significant position for treating adults with sepsis. However, the efficacy of corticosteroids when treating adults with sepsis is yet to be methodologically evaluated in a meta-analysis. Therefore, this study will perform an evaluation of the efficacy of corticosteroids in treating adult patients with sepsis. The results of this study could offer helpful evidence for clinical practice.

## Author contributions

**Conceptualization:** Wan-Peng Wang.

**Data curation:** Yan-Yan Zeng, Wan-Peng Wang.

**Formal analysis:** kepeng li, Li-Li Li.

**Funding acquisition:** Yan-Yan Zeng, Wan-Peng Wang.

**Methodology:** Yan-Yan Zeng, Li-Li Li, Wan-Peng Wang.

**Resources:** Li-Li Li.

**Software:** kepeng li, Yan-Yan Zeng, Wan-Peng Wang.

**Supervision:** Li-Li Li.

**Validation:** kepeng li, Yan-Yan Zeng, Li-Li Li.

**Writing – original draft:** kepeng li.

**Writing – review & editing:** kepeng li.
